# Effects of volatile organic compound ether on cell responses and gene expressions in *Arabidopsis*

**DOI:** 10.1186/s40529-015-0112-8

**Published:** 2016-01-06

**Authors:** Yun-Ting Tseng, Kuo-Chih Lin

**Affiliations:** Department of Life Science, National Dong Hwa University, Hualien County, 974 Taiwan

**Keywords:** Ether, Volatile organic compound, Ozone, Reactive oxygen species, Pollutant

## Abstract

**Background:**

The volatile organic compound ether is widely used as an industrial solvent and easily released to the environment. Our previous research indicated that ether triggers reactive oxygen species (ROS) production and activates ethylene biosynthetic genes and defense gene expressions in tomato. In the present study, we investigated the effect of ether on cell responses and gene expressions in *Arabidopsis* and compared the ROS and phytohormones produced in *Arabidopsis* and tomato plants in response to different air pollutants (O_3_ vs. ether).

**Results:**

Ether induced the sequential production of superoxide anion and hydrogen peroxide in *Arabidopsis*. Ether also triggered expressions of ethylene, salicylic acid and jasmonic acid biosynthetic genes. The temporal expression patterns of MAP kinase and protein phosphatase genes are in good accordance with those of the ethylene and salicylic acid biosynthetic genes, suggesting that induction of these phytohormone biosynthesis were through signaling pathways including both phosphorylation and/or dephosphorylation. By contrast, expression pattern of protein phosphatase PP2A3&4 coincided well with the expression of jasmonic acid biosynthetic gene LOX4, suggesting that induction of jasmonic acid biosynthesis is through PP2A3&4. However, the production of ROS and temporal expression patterns of phytohormone biosynthetic genes in *Arabidopsis* in response to ether were different from those to O_3_ and were different from those in tomato as well.

**Conclusions:**

Different plants have different strategies to respond to the same abiotic stress, and each plant species possesses its own unique signaling pathways that regulate the responding process.

**Electronic supplementary material:**

The online version of this article (doi:10.1186/s40529-015-0112-8) contains supplementary material, which is available to authorized users.

## Background

The deleterious effects of industrial air pollution on plants have been an issue due to the massive increase in fossil fuel combustion. Among the air pollutants, tropospheric ozone (O_3_) is considered the most phytotoxic, and there are many literature reports document the adverse effects of tropospheric O_3_ on crop yield, forest growth, and the metabolism and physiology of plants (Karnosky et al. 2007). In addition to ozone, the role of volatile organic compounds (VOC) as air pollutants has drawn much attention (Cape 2003). VOCs are carbon-based compounds that may contain oxygen, nitrogen, bromide and chloride atom and easily evaporate into the environment. The major sources of anthropogenic airborne VOCs are industrial processes, oil refining, fuel combustions, and vehicle exhaust emissions. Experimental studies have shown that some VOCs (e.g. acetate, formaldehyde, peroxyacetyl nitrate, and trichloroethylene.) are detrimental to vegetation, although most experiments investigated short-term exposures at high concentrations as opposed to ambient air conditions (Cape 2003). Cape (2003) prepared a review article, summarizing several studies on the effects of VOCs on vegetation at low concentrations, and long exposure times. VOCs may cause detrimental effects on leaves, flowering, seed production, protein content, plant metabolism, and total dry weight. Some countries publish emission inventories of VOCs, and take efforts to minimize VOC release into the environment. Despite a large body of research on the effects of O_3_ on plants at molecular and cellular levels, few reports describe the effects of VOCs on plants at the molecular and cellular levels.

For a period of time, the plants in our laboratory were polluted by diethyl ether (ether) that came from nearby chemical laboratory. Ether, a VOC, is widely used as an industrial solvent for waxes, fats, oils, perfumes, alkaloids, gums, and nitrocellulose, and also a solvent for hormone extraction from plant and animal tissues (Howard 1993). During its use as an industrial solvent and extractant, ether would easily release into the environment and evaporate into the atmosphere. Once in the vapor phase, it has very low chemical reactivity. Not absorbing light with wavelength longer than 290 nm, ether is not decomposed through photolysis (Howard 1993). However, ether can be photooxidized via vapor-phase reaction with photochemically produced hydroxyl radicals at a half-life of 29 h. Ether is not hydrolyzed or biodegraded in environmental media, so is persistent if released to soil or water. The compound is detectable in drinking water at water treatment plants, in the tap water in three out of ten North American cities, in drinking-water groundwater wells located downstream of municipal and industrial solid-waste landfill, and in the effluent of sewage treatment plants and chemical industries (Howard 1993). The detected concentrations range from 2.5–1000 μg/L.

In our previous research, we discovered that ether triggers a sequential production of superoxide anion (^·^O_2_^−^) and hydrogen peroxide (H_2_O_2_), and specifically induces tomato 1-aminocyclopropane-1-carboxylic acid oxidase (LeACO1) and LeACO4 genes through distinct signaling pathways (Lin et al. 2011). While the LeACO1 gene was induced via protein phosphorylation (e.g. via MAP kinase), the LeACO4 was induced via protein dephosphorylation (e.g. via protein phosphatase). Furthermore, induction of ACO gene expression occurred simultaneously with ROS accumulation and coincided with the occurrence of cell death. Our study results suggest that the cellular responses of tomato plants to ether are different from the plant responses to O_3_, and that tomato plants respond to different air pollutants through different perceptions and downstream signaling pathways (Lin et al. 2011). These findings unveiled the complex signaling networks of plant response to different air pollutants.

The purpose of the present study is to elucidate how the model plant *Arabidopsis* responds to ether in the context of ROS production and phytohormone biosynthetic gene expressions. The results will be helpful in understanding how differently plants (tomato vs. *Arabidopsis*) respond to the same type of molecule, ether, and how plants respond to different air pollutants (O_3_ vs. ether).

## Methods

### Plant materials

*Arabidopsis* seeds (*A. thaliana* cv Columbia) were soaked in ddH_2_O at 4 °C for 3 days and sown in wet soil. Seven days later, the seedlings were transferred to pots and grown in a temperature- and lighting-controlled growth chamber at 22 °C at 16/8 h light/dark cycles. Twenty-five- to thirty-day old plants were used for all the experiments.

### Ether fumigation and histochemical staining of ^·^O_2_^−^, H_2_O_2_, and dead cells

For ether fumigation, twenty-five- to thirty-day old plants were exposed to ether (Sigma, MO, USA) with 500 μL/L in a 4 L can for various time intervals. Histochemical staining of ^**·**^O_2_^−^, H_2_O_2_, and dead cells were performed as described by Lin et al. (2011). Two *Arabidopsis* plants were exposed to 500 μL/L ether fumigation for each time point, and all the mature leaves were harvested for histochemical staining. The ^**·**^O_2_^−^ production was detected by nitroblue tetrazolium (NBT) staining. The leaves were vacuum-infiltrated with 50 mL staining buffer [10 mM NaN_3_, 10 mM potassium phosphate buffer, pH7.8, and 0.1 % NBT] (Sigma, MO, USA) for 1 min and with a total of four times. The leaves were then incubated in the staining buffer in dark for 30 min. After staining, the leaves were cleared in boiling 70 % ethanol for 15 min. Production of ^**·**^O_2_^−^ was directly visualized by forming blue formazan precipitate. The H_2_O_2_ production was detected by the 3,3′-diaminobenzidine tetrahydrochloride (DAB) staining. The whole plants were removed from soil and soaked in 100 mL DAB staining buffer [10 mM 2-(*N*-morpholino)-ethane sulfonic acid (MES), pH6.5, and 0.1 % DAB] (Sigma, MO, USA) for 10 h in the dark. Following staining, the leaves were cleared in boiling 70 % ethanol for 15 min. Production of H_2_O_2_ was visualized as a reddish-brown coloration. Dead cells in the mesophyll tissues were assessed by the Evans blue staining. The leaves were vacuum-infiltrated with 50 mL of 1 % (w/v) Evans blue (Sigma, MO, USA) staining buffer for 1 min with a total of four times and then incubated in the staining buffer in dark for 30 min. After staining, the leaves were cleared in boiling 70 % ethanol for 5 min. Dead cells were unable to exclude Evans blue and were stained deep blue. The samples were mounted on glass slides with 30 % glycerol and examined with a fluorescence microscope (Eclipse E600, Nikon, Japan). The mechanically wounded plants that were punched with a brush leaving 0.5–1 mm holes spaced 2 mm apart were used a positive control. The density of the brush hole is approximately 25 holes/cm^2^. The wounded plants were then incubated in growth chamber for 3 h before staining.

### RNA extraction and real-time reverse transcription PCR (RT-PCR)

For RNA extraction, two plants were used for each time point and five to six mature leaves were collected. Total RNA was isolated from treated leaves using TRIzol™ reagent (Ambion, CA, USA) according to the manufacture’s protocol. Briefly, approximately 100 mg of leaves were homogenized with 1 mL of TRIzol reagent with a pestle. Two hundred microliters of chloroform were added to separate the RNA from cell pellets. The RNA was precipitated with 0.5 mL isopropanol. The RNA precipitates were washed with 70 % ethanol, air-dried, and resuspended with 20 μL DEPC-treated ddH_2_O. The concentration of RNA was determined by spectrophotometer. For cDNA synthesis, reverse transcription was performed in a 30 μL reaction volume containing 3 μg of the total RNA, 100 units of MMLV reverse transcriptase (Promega, WI, USA), 1X MMLV reaction buffer, 0.1 mM each of dNTPs (Thermo Scientific, MA, USA), and 1 μΜ oligo dT_18_ primer (Protech, Taipei, Taiwan). The reaction was carried out at 37 °C for 2 h. Real-time RT-PCR amplification of the cDNA clones were performed in a 10 μL reaction volume containing 2 μg of cDNA, 1X GoTaq™ qPCR Master Mix reagent (Promega, WI, USA), 1X CXR reference dye, and 0.2 μM of each primer (Additional file 1: Table S1). Thermocycling conditions were 95 °C for 2 min followed by 39 cycles of 95 °C for 15 s, 55 °C for 45 s, and 72 °C for 30 s, and a final 5 min at 72 °C on a CFX96™ real-time PCR detection system (Bio-Rad, USA). The amplified *Arabidopsis* *Actin2* (ACT2) was used as an internal control. Relative gene expression levels were calculated with the 2^−△△Ct^ method. Each value was the mean ± standard deviation of three independent experiments. The results were analyzed using Student’s *t* test. Differences of relative fold increase between the 0 min the other time points were considered statistically significant if *P* < 0.05 (*).

## Results

### Detection of cell death and ROS generation in response to ether

**Fig. 1:**
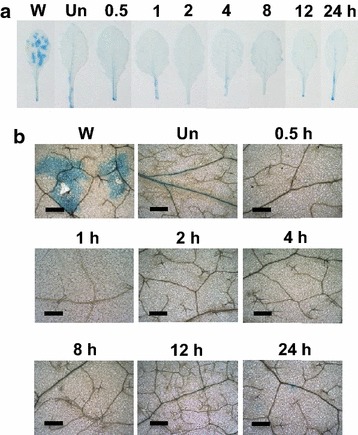
*Evans* *blue* assay for cell death caused by ether on *Arabidopsis* leaves. *Arabidopsis* plants were fumigated with 500 μL/L of ether for various time intervals (**a**). Panel (**b**) is the close-up pictures of the *Evans blue* staining. Three replicates for each treatment were performed, and representative leaves were presented. *W* wounding; *Un* untreated plants. *Scale bars* = 300 μm

**Fig. 2:**
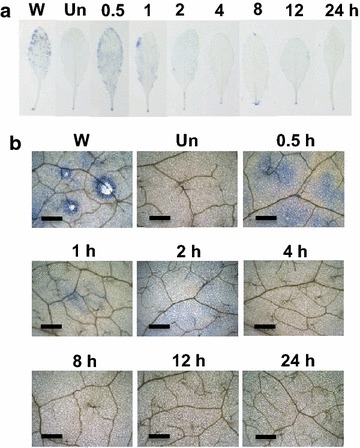
NBT assay for O_2_^−^ production induced by ether on *Arabidopsis* leaves. *Arabidopsis* plants were fumigated with 500 μL/L of ether for various time intervals (**a**). Panel (**b**) is the close-up pictures of the NBT stainings. Three replicates for each treatment were performed, and representative leaves were presented. *W* wounding; *Un* untreated plants. *Scale bars* = 300 μm

**Fig. 3:**
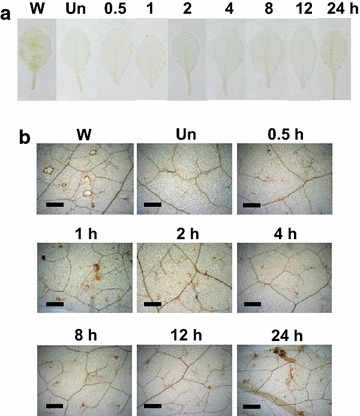
DAB assay for H_2_O_2_ production induced by ether on *Arabidopsis* leaves. *Arabidopsis* plants were fumigated with 500 μL/L of ether for various time intervals (**a**). Panel (**b**) is the close-up pictures of the DAB staining. Three replicates for each treatment were performed, and representative leaves were presented. *W* wounding; *Un* untreated plants. *Scale bars* = 300 μm

In an attempt to understand *Arabidopsis* plant response to acute exposure to ether, twenty-five- to thirty-day old *Arabidopsis* plants were exposed to ether fumigation for 24 h. No obvious cell death occurred during the 24 h of ether exposure (Fig. 1). To elucidate whether ether could induce cellular ROS (e.g. ^·^O_2_^−^ and H_2_O_2_) production, *Arabidopsis* plants were exposed to ether for various time intervals and histochemically stained with nitroblue tetrazolium (NBT) and 3,3′-diaminobenzidine tetrahydrochloride (DAB) to detect the accumulation of ^·^O_2_^−^ and H_2_O_2_, respectively. We detected a burst of ^·^O_2_^−^ production at 30 min after initiation of ether fumigation, and the amount decreased at 1 h and kept decreasing to a scarce level at 2 h (Fig. 2). No ^·^O_2_^−^ production was detectable thereafter. We detected a trace amount of H_2_O_2_ at 30 min, and a burst of H_2_O_2_ production was observed during 1–2 h of exposure (Fig. 3). The accumulation of H_2_O_2_ declined at 4 h, and the H_2_O_2_ levels remained low till 12 h. However, a small rise of H_2_O_2_ production was detectable after 24 h exposure to ether.

### Expressions of ROS producing and phytohormone biosynthetic genes in response to ether

**Fig. 4:**
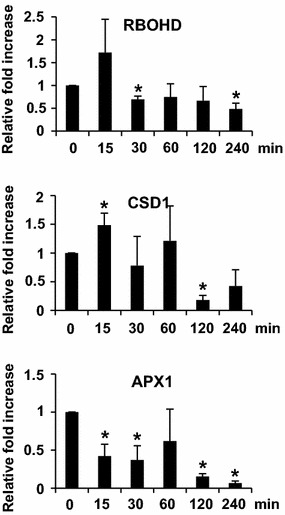
Real-time RT-PCR assay of ROS metabolizing gene expressions in response to ether. The relative fold increase of each gene was the average of three independent repeats

To further investigate the oxidative burst reactions in *Arabidopsis* in response to ether, we studied the temporal expression patterns of genes involved in ROS production. The selected genes were the NADPH oxidase (RBOHD), a gene responsible for catalyzing O_2_ to ^·^O_2_^−^, copper/zinc superoxide dismutase 1 (CSD1), a gene responsible for reducing ^·^O_2_^−^ to H_2_O_2_, and L-ascorbate peroxidase 1 (APX1) which involves in H_2_O_2_ reduction reaction (Overmyer et al. 2003). The results in Fig. 4 showed that the transcript levels of RBOHD increased at 15 min and then quickly decreased to the basal level at 30 min and thereafter. The transcript levels of CSD1 increased at 15–60 min and then decreased to the basal level thereafter. The transcript levels of APX1 showed a decreasing pattern over time.

**Fig. 5:**
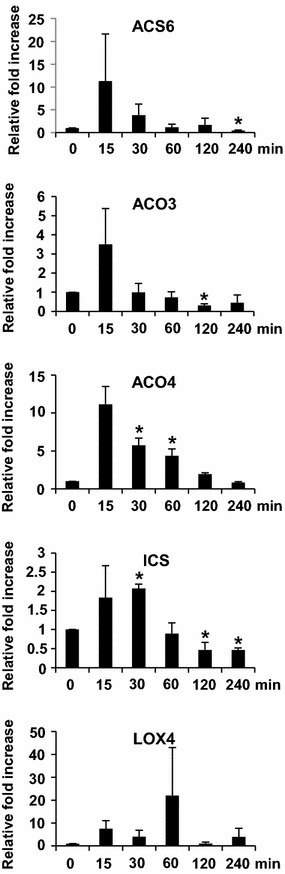
Real-time RT-PCR assay of hormone biosynthetic gene expressions in response to ether. The relative fold increase of each gene was the average of three independent repeats

To investigate the phytohormone biosynthesis in *Arabidopsis* in response to ether fumigation, we studied the temporal expression patterns of genes involved in biosynthesis of ethylene, salicylic acid, and jasmonic acid (Fig. 5). The selected genes were 1-aminocyclopropane-1-carboxylate synthase (ACS), 1-aminoocyclopropane-1-carboxylate oxidase (ACO), isochorismate synthase (ICS), and lipoxygenase (LOX) (Tamaoki 2008). ACS and ACO are responsible for ethylene biosynthesis. ACS converts S-adenosyl-methionine to 1-aminocyclopropane-1-carboxlic acid, and ACO subsequently converts 1-aminocyclopropane-1-carboxlic acid to ethylene. ICS, a key enzyme in salicylic acid biosynthesis, converts chorismate to isochorismate. LOX is a gene responsible for converting linolenic acids into 13-hydroperoxy-octadecatrienoic acid in the jasmonic acid biosynthetic pathway. Two ACS gene members, ACS2 and ACS6, which are activated in *Arabidopsis* in response to O_3_ were analyzed (Tosti et al. 2006). We detected no ACS2 transcript signal from ether-treated plants (data not shown). By contrast, the ACS6 transcripts greatly accumulated at 15 min, fell to the basal level at 60 min, and decreased thereafter. We designed gene-specific primer sets for analyzing the temporal expression patterns of ACO1**–**ACO5 genes in response to ether. Of these genes, we detected no signal for the ACO1 and ACO5 transcripts, and the transcript levels of ACO2 showed a decreasing pattern over time (data not shown). However, the ACO3 transcripts rapidly accumulated at 15 min and then quickly decreased to the basal level at 30 min. The ACO4 transcripts promptly accumulated at 15 min and then gradually decreased to basal level at 240 min. The transcript levels of ICS increased at 15 min and reached a maximum by 30 min. The transcript levels then started decreasing during the rest of exposure period. We chose LOX4 and LOX5, each belongs to the lipoxygenase gene family of distinct enzymatic activity groups, for analyzing their expression upon ether treatment (Bannenberg et al. 2008). The accumulation level of LOX4 transcripts exhibited a biphasic pattern. The transcript levels showed a quick rise at 15 min, reached a peak at 60 min, and declined to basal level by 120 min. The second peak was detected at 240 min post-exposure. We detected no signal for the LOX5 transcripts upon ether exposure (data not shown).

**Fig. 6:**
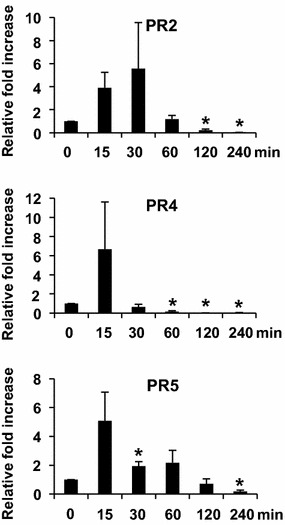
Real-time RT-CR assay of pathogenesis-related protein gene expressions in response to ether. The relative fold increase of each gene was the average of three independent repeats

Meanwhile, we determined the expression patterns of the pathogenesis-related protein (PR) 1, PR2, PR3, PR4, and PR5 in response to ether. We detected no PR1 and PR3 transcript signal from ether-treated plants (data not shown). The transcript levels of PR2 increased at 15 min, reached a maximal level by 30 min, and then decreased to basal level at 60 min (Fig. 6). The transcript levels of PR4 and PR5 increased at 15 min and then quickly decreased to basal level at 30 min after exposure.

### Determination of signaling pathways in response to ether

**Fig. 7:**
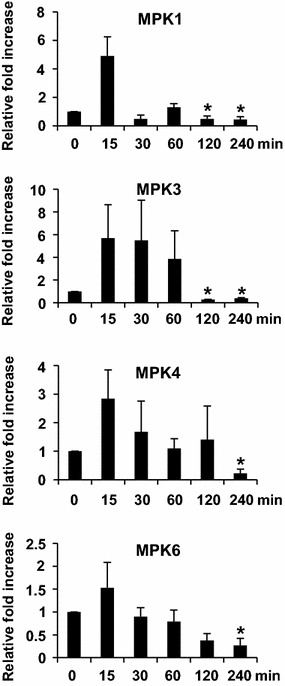
Assay for ether responsive phosphorylation signaling pathways with real-time RT-PCR. The relative fold increase of each gene was the average of three independent repeats

To elucidate whether protein phosphorylation and/or dephosphorylation might be involved in ether-induced signaling pathway, we investigated the temporal expression patterns of protein kinase and protein phosphatase genes in response to ether fumigation by real time RT-PCR. The protein kinase genes include mitogen-activating protein kinase (MPK) 1, MPK3, MPK4, and MPK6 which are activated in response to O_3_ and other abiotic stresses (Ahlfors et al. 2004; Mishra et al. 2006). The results in Fig. 7 revealed that MPK1 and MPK6 exhibited similar transcript accumulation patterns, which are quickly accumulated at 15 min and dropped to basal level at 30 min and thereafter. Increasing levels of MPK3 transcripts appeared at 15–60 min, and then dropped to basal level thereafter. The transcript levels of MPK4 increased at 15–30 min and dropped to basal level at 60 min and thereafter.

**Fig. 8:**
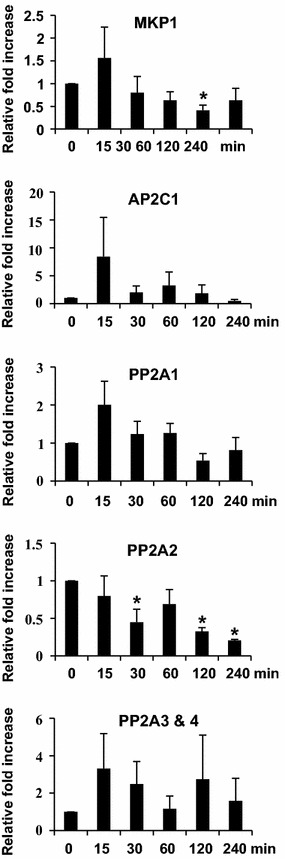
Assay for ether responsive dephosphorylation signaling pathways with real-time RT-PCR. The relative fold increase of each gene was the average of three independent repeats

We studied the expression patterns of protein phosphatase genes in response to ether by real time RT-PCR. These genes include MAP kinase phosphatase 1 (MKP1), protein phosphatase (PP) 2A1 (PP2A1), PP2A2, PP2A3&4, and PP2C-type phosphatase AP2C1 (AP2C1). These phosphatases are involved in modulating phytohormone biosynthesis in response to ozone or developmental cues (Ludwików et al. 2014; Schweighofer et al. 2007; Skottke et al. 2011). The results in Fig. 8 revealed that the transcript accumulation patterns of MKP1, AP2C1, and PP2A1 were similar. Their transcript levels accumulated at 15 min and then gradually decreased to basal level by 30 or 120 min post-exposure. PP2A2 showed a straight decreasing pattern during the ether fumigation period. By contrast, the transcript accumulation of PP2A3&4 exhibited a biphasic pattern. The first peak was present at 15 min and declined to basal level by 60 min. The second peak was detectable at 120 min post-exposure and returned to basal level by 240 min.

## Discussion

**Fig. 9:**
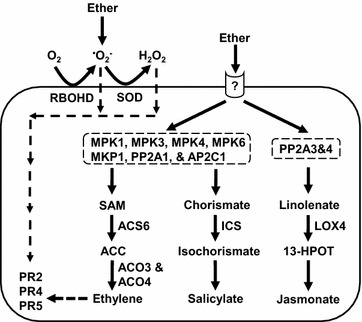
A schematic model illustrating the responses and signaling pathways for activation of gene expressions in response to ether. The *dashed boxes* indicate possible kinase and phosphatase involved. The *dashed lines* indicate the possible involvement of ethylene and ROS on PR protein gene induction

Base on the observations of ROS production, ROS and phytohormone biosynthetic gene expressions, PR gene expressions and signaling gene expressions, we proposed a model for depicting the responses of *Arabidopsis* to ether fumigation (Fig. 9).

### Oxidative burst is a general response of plants to different air pollutants

Oxidative burst is a general response of sensitive plants to air pollutant O_3_ (Wohlgemuth et al. 2002). In tomato and tobacco, H_2_O_2_ is the major ROS produced in response to O_3_, and ethylene is involved in the amplification of ROS production and regulation of cell death (Moeder et al. 2002; Schraudner et al. 1998; Castagna et al. 2007). By contrast, ^·^O_2_^−^ is the major ROS produced in *Arabidopsis* in response to O_3_, and salicylic acid is responsible for cell death (Rao and Davis 1999). Our previous study showed that acute exposure of tomato plants to ether causes cell death and that ^·^O_2_^−^ was the major ROS produced in response to ether (Lin et al. 2011). In the present study, we detected a transient, sequential production of ^·^O_2_^−^ and H_2_O_2_ in *Arabidopsis* in response to ether, and no cell death observed, suggesting that *Arabidopsis* is not as susceptible to ether treatment as tomato is (Figs. 1, 2, 3). Expression patterns of ROS metabolizing genes such as RBOHD and CSD1 corresponded well with the histochemical observations of ROS accumulation. Production of RBOHD transcripts at 15 min after initiation of ether fumigation coincided well with the accumulation of ^·^O_2_^−^ at 30 min post-exposure (Figs. 2, 4). Diminution of ^·^O_2_^−^ at 30–60 min was accompanied by accumulation of H_2_O_2_ at 30–120 min (Figs. 2, 3). Furthermore, induction of CSD1 at 15–60 min was also in good accordance with the production of H_2_O_2_ (Figs. 3, 4). All these results suggested that ^·^O_2_^−^ and H_2_O_2_ were sequentially produced by RBOHD and CSD, respectively, in *Arabidopsis* in response to ether, and oxidative burst is a general response of plants to different air pollutants (Fig. 9).

Ascorbate is believed to provide important protection in the apoplast against O_3_-derived ROS (Castagna and Ranieri, 2009). In addition to direct reaction with ROS, ascorbate also acts as a reducing substrate for ascorbate peroxidase (APX), which scavenges H_2_O_2_ and thus affects the propagation of the O_3_ stress. However, in this study no induction of APX1 gene expression in ether-treated *Arabidopsis* suggested that ether-derived ROS were more likely to be quickly quenched by direct reaction with ascorbate or other antioxidant systems rather than by APX1 (Fig. 4).

### Induction of salicylate and ethylene accumulation by ether does not suppress jasmonate biosynthesis

In O_3_-treated *Arabidopsis*, salicylic acid is the key signal in antioxidant response and in initiating O_3_-induced cell death in *Arabidopsis* (Rao and Davis 1999; Tamaoki 2008). O_3_ stimulates the production of ethylene via salicylic acid. Both salicylic acid and ethylene suppress jasmonic acid biosynthesis and act in concert to induce high levels of ^·^O_2_^−^ or ^·^O_2_^−^ together with H_2_O_2_, and the production of ROS finally leads to cell death (Rao and Davis 1999; Wohlgemuth et al. 2002). By contrast, in O_3_-treated tomato ethylene is the key factor that triggers the progression of O_3_-induced lesions (Castagna et al. 2007). Furthermore, O_3_ induce ethylene and jasmonate biosynthesis, although with different timings and to different extents. In the present study, ether stimulated expressions of salicylic biosynthetic gene, ICS, transcripts from 15–30 min, and the amount returned to basal level by 60 min post-exposure (Fig. 5). The transcript levels of ethylene biosynthetic genes, ACS6, ACO3, and ACO4, were also promptly accumulated at 15 min and began to decrease at 30 min (Fig. 5). Occurrence of ROS production and ethylene and salicylic acid biosynthesis at the very early stage of ether treatment suggested that these three events simultaneously occurred in response to ether (Figs. 2, 3, 4, 5). Furthermore, no cell death occurred during this time period or thereafter, suggesting that production of ROS and ethylene and salicylic acid biosynthesis in response to ether did not promote cell death as they do in *Arabidopsis* in response to O_3_.

We also detected an accumulation of jasmonic acid biosynthetic gene transcripts, LOX4, following exposure to ether for 15 min and maintaining at high level till 240 min, suggesting that ether also promptly induces jasmonic acid biosynthesis (Fig. 5). However, continuous induction of LOX4 gene at the time that ICS, ACS6, ACO3, and ACO4 gene expressions decreased in response to ether indicated that induction of salicylic acid and ethylene did not suppress the jasmonic acid biosynthesis as they do in response to O_3_ (Rao and Davis 1999; Wohlgemuth et al. 2002). Biosynthesis of jasmonic acid may inhibit the spread of cell death (Tuominen et al. 2004). Therefore, it is possible that continuous production of jasmonic acid suppressed the occurrence and spread of cell death during ether exposure.

We assayed five PR genes and found that the expression patterns of PR2, PR4, and PR5 were similar to those of the ethylene and salicylic acid biosynthetic genes (Figs. 5, 6). Expressions of SA-dependent PR1 and ethylene-dependent PR4 genes in *Arabidopsis* have been reported to be induced under O_3_ stress (Rao et al. 2002). The result of our previous study suggested that ethylene produced in tomato plants in response to ether activates expression of PR4 but not PR1 genes (Lin et al. 2011). Therefore, it is possible that expressions of these PR genes were induced by ethylene in *Arabidopsis* in response to ether (Fig. 9, dash lines). In addition, low dosage exposure to ROS may produce signaling effects on growth, development, stress acclimation, and pathogen responses (Foyer and Noctor 2005). Therefore, ether-generated ROS may play a role in signaling modulation and induce the expression of the PR genes (Fig. 9, dash lines). However, whether expressions of the PR genes were induced by ethylene or ROS signaling during ether fumigation remains unclear and needs further elucidation.

### The ether responsive signaling pathways

The signal transduction pathways induced by O_3_ exposure have been extensively elucidated in *Arabidopsis*, whereas rare reports, if any, are focused on tomato. MPK3 and MPK6 in *Arabidopsis* are activated within 30–60 min in response to O_3_ (Ahlfors et al. 2004). Phosphorylation of ACS2 and ACS6 by MPK3 and MPK6 results in increased ethylene biosynthesis through protein stabilization (Liu and Zhang 2004; Han et al. 2010). Skottke et al. (2011) showed that PP2A mediates a finely tuned regulation of overall ethylene production by differentially affecting the stability of specific classes of ACS enzymes. Ludwików et al. (2014) showed that the *Arabidopsis* protein phosphatase type 2C is involved in the regulation of ethylene biosynthesis during ozone stress. In this study, the temporal expression patterns of MAP kinase and protein phosphatase genes were in good accordance with those of the hormone biosynthetic genes. The temporal expression patterns of ACS6, ACO3, ACO4 and ICS were in unanimity with the expression patterns of MAP kinase, MPK1, MPK3, MPK4, and MPK6, and protein phosphatase, MKP1, AP2C1, and PP2A1 (Figs. 5, 7, 8). They were all induced at 15–60 min, suggesting that MPK1, MPK3, MPK4, MPK6, MKP1, AP2C1, and PP2A1 were involved in protein phosphorylation/dephosphorylation leading to induction of ethylene and salicylic acid biosynthesis by ether (Fig. 9). Expression patterns of protein phosphatase PP2A3&4 coincided well with the expression of LOX4 (Figs. 5, 8). Both LOX4 and PP2A3&4 transcripts exhibited a biphasic accumulation pattern. Their transcript levels showed a quick rise at 15 min and reached a peak at 15–60 min, and the second peak was detected at 120–240 min post-exposure. The sequential expression of PP2A3&4 and LOX4 genes suggested that induction of jasmonic acid biosynthesis might be through phosphatase PP2A3&4 (Fig. 9). Although further experiments are required to confirm our model, ether induction of MAP kinase and protein phosphatase genes reinforces the idea that signaling events initiated by diverse biotic or abiotic stresses may converge at the point of MAP kinases and/or phosphatase (Farkas et al. 2007; Zhang et al. 2006).

## Conclusion

In this study, ^·^O_2_^−^ and H_2_O_2_ were sequentially produced in *Arabidopsis* in response to ether, indicating that oxidative burst is a general response of plants to different air pollutants. Induction of salicylic acid and ethylene did not suppress the jasmonic acid biosynthesis as they do in response to O_3_ treatment, suggesting that plant responds differently to different air pollutants. The different ROS and hormones produced in *Arabidopsis* and tomato plants in response to different air pollutants (O3 vs. ether) suggested that different plants have different strategies to respond to the same abiotic stress, and each plant species possesses its own unique signaling pathways that regulate the responding process.

## Additional file

**Additional file 1: Table S1. ** The primers used for real-time RT-PCR assay.
