# Supplementary material for: Effects of volatile organic compound ether on cell responses and gene expressions in Arabidopsis
Source: Bot Stud. 2016 Jan 6;57:1. doi: 10.1186/s40529-015-0112-8 (PMC5430555; doi:10.1186/s40529-015-0112-8)
Supplement: Supplementary file 1 — Additional file 1: Table S1. The primers used for real-time RT-PCR assay. [file 40529_2015_112_MOESM1_ESM.pdf]

Supplementary information. The primers used for real-time RT-PCR assay.

Gene	Accession number	Forward primer sequence 5’ -> 3’	Reverse primer sequence 5’ -> 3’
RBOHD	NM_124165	^{1441}TCGAAACACCATCACTTGGCT	^{1565}CCGCATGGAGTAAAACACCG
CSD1	NM_100757	^{124}TGTTACGGGGACTATCTTTT	^{276} TTGAAATGTGGACCAGTAGA
APX1	NM_001035914	^{285}ATGACGAAGAACTACCCAAC	^{575}ATGGAAATCAGCAAAAGAGA
ACS 2	NM_100030	^{1050}TCCGATGATCAGTTTGTGGA	^{1293}AGGAAGAGCCAGGAGACACA
ACS 6	NM_117199	^{231}ATGGGTCTCGCTGAAAATCA	^{579}TCACTCCGGTTCATCTC
ACO1	NM_127517	^{29}GCAACTATGCCTCCTAACAC	^{217}CTTCTCTCCATCCAACTCTG
ACO2	NM_104918	^{657}AGCAATTATCCACCATGTCC	^{856}TTGGTTATCACCTCAAGTTG
ACO3	NM_101073	^{224}GAGAGGATGACAAAGGAACA	^{371}TAGATTGAGGGAGATGATGG
ACO4	NM_100380	^{774}TCGATGTTCCTCCGGTTAAG	^{977}CTGCTTCTTTTCCGATCAGC
ACO5	NM_106161	^{456}AGCGAGCAAGATGATGGAAGT	^{603}AGGATGAGGACAAGGAGGGT
ICS	NM_106129 NM_101744	^{682}CCTCTGATTCGTGCCTATGG	^{1094}GGATCAATGTCGGTATCCGT
LOX4	NM_105911	^{1311}CGAGGGCTTGCTTAGATACG	^{1502}GTGAGAGCGGAGTGAAGACC
LOX5	NM_113137	^{1065}CTCTGTTTGCGACAAGACCA	^{1273}CCGATCTGCTCTCTTTGAGG
PR1	NM_127025	^{77}GCTCTTGTAGGTGCTCTTGT	^{240}TTGCCTCTTAGTTGTTCTGC
PR2	NM_115586	^{538}TCCTCCGTCTCAAGGAAGGT	^{668}CCGTGTCTCCCATGTAGCTG
PR3	NM_112085	^{3}TGCCTCCACAAAAAGAAAACCA	^{149}CATAGACCGTTGGGGCAGAG
PR4	NM_111344	^{211}CAATTGTTGGGGAAGTGGGC	^{326}AAGCACTCACGGCTCTCAAA
PR5	NM_106161	^{325}GGAGACTGTGGCGGTCTAAG	^{472}CGGATGGTCTTATCCCCAGC
MPK1	NM_100895	^{863}TCGGAAGCCAAAGAGAAGAA	^{1069}TGTATGGATGCTGGAGTGCT
MPK3	NM_114433	^{810}ATGTTTGGTCTGTTGGTTGT	^{969}TATCTTTTCGCATCCTCATT
MPK4	NM_116367	^{1215}ACCGTGAAACAGTCAAGTTC	^{1327}AAAGCAGAGCAAAACAAAAC
MPK6	NM_129941	^{766}CAAACTGCGACCTAAAAATC	^{927}TACAGCCTACAGACCAAACA
MKP1	NM_115385	^{2032}TCCCTTCCCAAACTTCTCCT	^{2194}AAAGCAGGTGTGCCTCTGTT
AP2C1	NM_128557	^{132}GACCTCTTCTCCTTCCTCTC	^{415}CATCACCTTCTCTCTCAACC
PP2A1	NM_104676	^{492}AAGGTGCGTTACAGGGACAGA	^{595}CCGTATTTCCTCAAGCATTCG
PP2A2	NM_100918	^{254}TATCGGAGGCGGATGTGAG	^{362}GAATATCGCCGCAAACGGTA
PP2A3 & 4	NM_001202803	^{499}CTGTTACGCTGTTAGTCGCC	^{634}TTGCGTTGCCGTACTTTCGT
ACT2	NM_112764	^{638}AACTCTCCCGCTATGTATGTCGC	^{938}CCATCTCCTGCTCGTAGTCAACA
